# Evaluation of Autof MS 1000 and Vitek MS MALDI-TOF MS System in Identification of Closely-Related Yeasts Causing Invasive Fungal Diseases

**DOI:** 10.3389/fcimb.2021.628828

**Published:** 2021-02-18

**Authors:** Qiaolian Yi, Meng Xiao, Xin Fan, Ge Zhang, Yang Yang, Jing-Jia Zhang, Si-Meng Duan, Jing-Wei Cheng, Ying Li, Meng-Lan Zhou, Shu-Ying Yu, Jing-Jing Huang, Xin-Fei Chen, Xin Hou, Fanrong Kong, Timothy Kudinha, Ying-Chun Xu

**Affiliations:** ^1^ Department of Laboratory Medicine, and Beijing Key Laboratory for Mechanisms Research and Precision Diagnosis of Invasive Fungal Diseases, Peking Union Medical College Hospital, Chinese Academy of Medical Sciences, Beijing, China; ^2^ Graduate School, Peking Union Medical College, Beijing, China; ^3^ Department of Infectious Diseases and Clinical Microbiology, Beijing Chaoyang Hospital, Capital Medical University, Beijing, China; ^4^ Department of Laboratory Medicine, Beijing Friendship Hospital, Capital Medical University, Beijing, China; ^5^ Centre for Infectious Diseases and Microbiology Laboratory Services, Institute of Clinical Pathology and Medical Research, New South Wales Health Pathology, Westmead Hospital, The University of Sydney, Westmead, NSW, Australia; ^6^ Department of Clinical Laboratory, Charles Sturt University, Orange, NSW, Australia; ^7^ New South Wales Health Pathology, Regional and Rural, Orange Hospital, NSW, Australia

**Keywords:** MALDI-TOF MS, closely-related yeasts, invasive fungal diseases, Autof MS 1000, Vitek MS

## Abstract

Matrix-assisted laser desorption ionization-time of flight mass spectrometry (MALDI-TOF MS) has been accepted as a rapid, accurate, and less labor-intensive method in the identification of microorganisms in clinical laboratories. However, there is limited data on systematic evaluation of its effectiveness in the identification of phylogenetically closely-related yeast species. In this study, we evaluated two commercially available MALDI-TOF systems, Autof MS 1000 and Vitek MS, for the identification of yeasts within closely-related species complexes. A total of 1,228 yeast isolates, representing 14 different species of five species complexes, including 479 of *Candida parapsilosis* complex, 323 of *Candida albicans* complex, 95 of *Candida glabrata* complex, 16 of *Candida haemulonii* complex (including two *Candida auris*), and 315 of *Cryptococcus neoformans* complex, collected under the National China Hospital Invasive Fungal Surveillance Net (CHIF-NET) program, were studied. Autof MS 1000 and Vitek MS systems correctly identified 99.2% and 89.2% of the isolates, with major error rate of 0.4% versus 1.6%, and minor error rate of 0.1% versus 3.5%, respectively. The proportion of isolates accurately identified by Autof MS 1000 and Vitek MS per each yeast complex, respectively, was as follows; *C. albicans* complex, 99.4% vs 96.3%; *C. parapsilosis* complex, 99.0% vs 79.1%; *C glabrata* complex, 98.9% vs 94.7%; *C. haemulonii* complex, 100% vs 93.8%; and *C. neoformans*, 99.4% vs 95.2%. Overall, Autof MS 1000 exhibited good capacity in yeast identification while Vitek MS had lower identification accuracy, especially in the identification of less common species within phylogenetically closely-related species complexes.

## Introduction

Invasive fungal diseases (IFD) have become an emerging healthcare problem worldwide. It is associated with high rates of morbidity and mortality in immunocompromised individuals and critically ill patients (Miceli et al., 2011; Lockhart et al., 2017). Rapid, reliable, and species-specific diagnosis of fungal pathogens causing IFD is a prerequisite for cost-effective and successful therapy. However, some closely-related yeast species complexes are difficult to identify by conventional morphological or biochemical methods, and several of these cryptic species have distinct antifungal susceptibility profiles associated with specific clinical settings. These include *Candida parapsilosis sensu stricto*, *Candida metapsilosis*, *Candida orthopsilosis*, *Lodderomyces elongisporus* of the *C. parapsilosis* complex, *Candida albicans* and *Candida dubliniensis* of the *C. albicans* complex, *Candida glabrata sensu stricto*, *Candida nivariensis*, and *Candida bracarensis* of the *C. glabrata* complex, *Cryptococcus neoformans*, and *Cryptococcus gattii* of the *C. neoformans* complex, and finally, *Candida haemulonii*, *Candida duobushaemulonii*, and *Candida auris* of the *C. haemulonii* complex (also refered as multidrug-resistant [MDR] complex) (Muñoz et al., 2018).

Within less than a decade, the introduction of MALDI-TOF MS has enabled rapid and accurate identification of microorganisms including bacteria, mycobacteria, yeasts and filamentous fungi in clinical laboratories (Odds et al., 2007; Rychert et al., 2018) Autof MS 1000 (Autobio Diagnostics, Zhengzhou, China), a commercial MALDI-TOF MS system, has been available for routine pathogen identification in many clinical laboratories in China since 2018 (Wang et al., 2019). However, its performance and application in the identification of yeasts has not been fully evaluated. The purpose of this study was to evaluate the accuracy of Autof MS 1000 and Vitek MS in the identification of yeasts causing IFDs, especially for pathogens within closely-related species complexes.

## Materials and Methods

### Yeast Isolates

A total of 1,228 yeast isolates causing IFDs, and collected from 57 hospitals in China during the period August 2016 to July 2017, under the CHIF-NET program, were studied. Isolates were initially inoculated on a chromogenic agar (Brilliance C., Oxoid Ltd., Hampshire, United Kingdom) then subcultured on Sabouraud dextrose agar (SDA), and incubated at 35°C for 24 to 48 h.

### Sequencing-Based Identification

All isolates were identified by DNA sequencing of the internal transcribed spacer (ITS) region, which was considered the “gold standard”. Primers ITS1 (5’-TCC GTA GGT GAA CCT GCG G-3’) and ITS4 (5’-TCC TCC GCT TAT TGA TAT GC-3’) were used to amplify the full-length ITS region. Amplification of the ITS region was carried out under the following conditions: denaturation at 94°C for 5 min, followed by 30 cycles of denaturation at 94°C for 30 s, annealing at 56°C for 90 s, and elongation at 72°C for 75 s, with a final extension step of 10 min at 72°C. Strain species identification was performed as described in a previous study (Wang et al., 2016).

### Vitek MS Identification

A thin smear of a freshly cultured isolate was deposited onto a target plate, and 1 μl of formic acid (70%) was added. After air-drying, each spot was overlaid with 1 μl of α-cyano-4-hydroxycinnamic acid (HCCA) matrix (bioMérieux). The matrix was then allowed to dry at room air. *E.coli* ATCC 8739 was utilized as a calibrant and quality control strain per acquisition group on each slide. Measurement was then performed following the manufacturer’s suggested setting using automated collecting spectra. Captured spectra were analyzed based on IVD database version 3.0. For data analysis, a “green frame” indicator with a reliability (probability) of between 60.0% and 99.9% indicated sufficient species level identification, and a “yellow triangle” indicator denoted low resolution. If no identification (NoID) was provided, the isolate was considered unidentified and presented as a “red circle”.

### Autof MS 1000 Identification

Autof MS 1000 is a new Chinese brand of MALDI-TOF MS platform for identification of micro-organisms which was approved for clinical use by China National Medical Products Administration in 2020. The operation process of this platform is similar to that of Vitek MS. A thin smear of a freshly cultured isolate was deposited onto a target plate, and 1 μl of formic acid (70%) was added. After air-drying, each spot was overlaid with 1 μl of α-cyano-4-hydroxycinnamic acid (HCCA) matrix. But without a fixed calibration point, Autof MS 1000 system runs identification immediately after sampling. The database (Autof Acquirer Version V2.0.18) contains 14,125 microbial strains comprising 4,226 species, 360 of which are fungal species. Results were interpreted based on the log score value of the first best match following manufacturer’s instructions as follows: 0.0≤score<6.0, not reliable identification; 6.0≤score<9.0, genus-level reliable identification and probable species-level identification; score≥9.0 species-level reliable identification.

### Criteria for Identification and Discrepant Analysis

In the case of discrepant results or no identification result for one or both methods, the result of ITS sequencing was considered the final correct identification. A major error (ME) in identification by each of the methods studied (compared to the gold standard ITS) was defined as the incorrect genus identification (“no identification” not included). A minor error (MIE) was defined as the correct genus identification with incorrect species identification.

## Results

### Correct Identification by Vitek MS and Autof MS 1000

A total of 1,228 isolates representing 14 different yeast species within five closely-related species complexes (ie. *C. albicans* complex, *C. parapsilosis* complex, *C. glabrata* complex, *C. haemulonii* complex and *C. neoformans* complex), were evaluated in this study. Out of these isolates, 1,218 (99.2%) were correctly identified to species level by the Autof MS 1000 system, while 1,095 (89.2%) were correctly identified by Vitek MS (**Table 1**).

**Table 1:** Results of matrix-assisted laser desorption ionization-time of flight mass spectrometry (MALDI-TOF MS) identification by Autof MS 1000 and Vitek MS.

Organism defined by ITS sequencing (no. of isolates)	Autof MS 1000								Vitek MS							
	Agree		ME		MIE		No ID		Agree		ME		MIE		No ID	
*C. parapsilosis* complex (n=479)	474	99.0%	2	0.4%	0	0.0%	3	0.6%	379	79.1%	9	1.9%	40	8.4%	51	10.6%
* C. parapsilosis* (384)	380	99.0%	1	0.3%	0	0.0%	3	0.8%	366	95.3%	3	0.8%	0	0.0%	15	3.9%
* C. metapsilosis* (64)	64	100.0%	0	0.0%	0	0.0%	0	0.0%	3	4.7%	5	7.8%	25	39.1%	31	48.4%
* C. orthopsilosis* (26)	25	96.2%	1	3.8%	0	0.0%	0	0.0%	6	23.1%	1	3.8%	15	57.7%	4	15.4%
* L. elongisporus* (5)	5	100.0%	0	0.0%	0	0.0%	0	0.0%	4	80.0%	0	0.0%	0	0.0%	1	20.0%
*C. albicans* complex (n=323)	321	99.4%	2	0.6%	0	0.0%	0	0.0%	311	96.3%	8	2.5%	0	0.0%	4	1.2%
* C. albicans* (322)	320	99.4%	2	0.6%	0	0.0%	0	0.0%	310	96.3%	8	2.5%	0	0.0%	4	1.2%
* C. dubliniensis* (1)	1	100.0%	0	0.0%	0	0.0%	0	0.0%	1	100.0%	0	0.0%	0	0.0%	0	0.0%
*C. neoformans* complex (n=315)	313	99.4%	0	0.0%	1	0.3%	1	0.3%	300	95.2%	2	0.6%	2	0.6%	11	3.5%
* C. neoformans* (305)	304	99.7%	0	0.0%	0	0.0%	1	0.3%	297	97.4%	2	0.7%	0	0.0%	6	2.0%
* C. gattii* (10)	9	90.0%	0	0.0%	1	10.0%	0	0.0%	3	30.0%	0	0.0%	2	20.0%	5	50.0%
*C. glabrata* complex (n=95)	94	98.9%	1	1.1%	0	0.0%	0	0.0%	90	94.7%	1	1.1%	1	1.1%	3	3.2%
* C. glabrata* (91)	90	98.9%	1	1.1%	0	0.0%	0	0.0%	90	98.9%	1	1.1%	0	0.0%	0	0.0%
* C. nivariensis* (3)	3	100.0%	0	0.0%	0	0.0%	0	0.0%	0	0.0%	0	0.0%	1	33.3%	2	66.7%
C. bracarensis* (1)	1	100.0%	0	0.0%	0	0.0%	0	0.0%	0	0.0%	0	0.0%	0	0.0%	1	100.0%
*C. haemulonii* complex (n=16)	16	100.0%	0	0.0%	0	0.0%	0	0.0%	15	93.8%	0	0.0%	0	0.0%	1	6.3%
* C. haemulonii* (11)	11	100.0%	0	0.0%	0	0.0%	0	0.0%	11	100.0%	0	0.0%	0	0.0%	0	0.0%
* C. duobushaemulonii* (3)	3	100.0%	0	0.0%	0	0.0%	0	0.0%	2	66.7%	0	0.0%	0	0.0%	1	33.3%
* C. auris* (2)	2	100.0%	0	0.0%	0	0.0%	0	0.0%	2	100.0%	0	0.0%	0	0.0%	0	0.0%
Total (1228)	1218	99.2%	5	0.4%	1	0.1%	4	0.3%	1095	89.2%	20	1.6%	43	3.5%	70	5.7%

ITS, internal transcribed spacer region; ME, major error; MIE, minor error; No ID, no identification.

*Candida bracarensis was not included in database IVD 3.0 of Vitek MS system.

A majority (96.3%, 311/323) of *C. albicans* complex isolates were correctly identified to the species level by Vitek MS. For Autof MS 1000, the identification accuracy rate for *C. albicans* complex isolates was 99.4% (321/323). For *C. glabrata* complex isolates, 98.9% (94/95) were correctly identified to the species level by Autof MS 1000, which was higher than that of Vitek MS at 94.7% (90/95). For *C. parapsilosis* complex, 79.1% (379/479) of the isolates were correctly identified to species level by Vitek MS, which was much less than that of Autof MS 1000 at 99.0% (474/479). For *C. neoformans* complex, 95.2% (300/315) were correctly identified to the species level by Vitek MS while for Autof MS 1000, 99.4% (313/315) were correctly identified to species level (**Table 1**).

### Comparison of “No Identification” Results

Four isolates (0.3%) yielded “no identification” result by Autos MS 1000, and 70 (5.7%) by Vitek MS (**Table 1**).

For species included in both systems’ identification databases, there were two (2/3, 66.6%) *C. nivariensis* isolates and 31 (31/64, 48.4%) of *C. metapsilosis* that were not identified by Vitek MS, which was a direct opposite to Autof MS 1000 which correctly identified both species. There were 5 (5/10, 50.0%) *C. gattii* isolates that were unidentified by Vitek MS.

In addition, *C. bracarensis* (n=1) was correctly identified by Autof MS 1000, but unidentified by Vitek MS as this species is not included in the Vitek MS database IVD 3.0.

### Misidentifications by Vitek MS and Autof MS 1000

Autof MS 1000 identification results of five isolates (0.4%) were defined as major error while for Vitek MS it was for 20 isolates (1.6%) (**Table 2**). Only one identification result from an isolate was defined as a minor error for Autof MS 1000, while for Vitek MS, there were 43 (3.5%) isolates whose identification results were considered minor errors. One *C. nivariensis* (n=3) isolate was misidentified as *C. glabrata*; in addition, among *C. metapsilosis* (n=64) isolates, the identification results of five isolates were considered as ME, and 25 as MIE by Vitek MS. For *C. gattii* (n=10) there was one identification result that was considered as MIE by Autof MS 1000, and two by Vitek MS.

**Table 2:** Misidentification and “no identification” results by two systems.

Species by ITS sequencing	Autof MS 1000	Vitek MS
*C. parapsilosis* complex (n=479)		
*C. parapsilosis* (384)	No ID (3) *C. guilliermondii* (1)	No ID (15) *C. albicans* (2) *C. pelliculosa* (1)
*C. orthopsilosis* (26)	*C. albicans* (1)	No ID (4) *C. parapsilosis* (15) *C. albicans* (1)
*C. metapsilosis* (64)	0	No ID (32) *C. parapsilosis* (15) *C. orthopsilosis* (5) *C. pelliculosa* (1) *C. pulcherrima* (2) *C. laurentii* (1) *L. elongisporus* (4) *P. italicum* (1)
*L. elongisporus* (5)	0	No ID (1)
*C. glabrata* complex (n=95)		
*C. glabrata* (91)	*C. guilliermondii* (1)	*C. parapsilosis* (1)
*C. nivariensis* (3)	0	No ID (2) *C. glabrata* (1)
*C. bracarensis* (1)	0	No ID (1)
*C. albicans* complex (n=323)		
*C. albicans* (322)	*C. parapsilosis* (1) *C. tropicalis* (1)	No ID (4) *C. utilis* (4) *C. parapsilosis* (1) *C. tropicalis* (1) *C. glabrata*/*C. tropicalis*/*C. famata* (1) *C. tropicalis*/*C. dubliniensis* (1)
*C. dubliniensis* (1)	0	0
*C. neoformans* complex (n=315)		
*C. neoformans* (305)	No ID (1)	No ID (6) *C. tropicalis* (1) *C. parapsilosis* (1)
*C. gattii* (10)	*C. neoformans* (1)	No ID (5) *C. neoformans* (2)
*C. haemulonii* complex (n=16)		
*C. duobushaemulonii* (3)	0	No ID (1)
*C. haemulonii* (11)	0	0
*C. auris* (2)	0	0

## Discussion

Accurate identification of pathogens is important to ensure accurate diagnosis of diseases and subsequent proper treatment of the condition. As a newly licensed MALDI-TOF MS platform, few comparisons have been done between Autof MS 1000 and other systems. Recent studies have evaluated the performance of Autof MS 1000 in the identification of *Bacteroides fragilis* group isolates (Wang et al., 2019) and clinically relevant filamentous fungi (Sun et al., 2020). In this study, we evaluated the performance of MALDI-TOF MS systems, Autof MS 1000 and Vitek MS, in the identification of closely-related yeast species causing IFDs, using ITS sequencing as the reference method. Previous studies have shown that the accuracy for yeast identification ranges from 76.5% to 96.2% for Vitek MS (Wang et al., 2016). In this study, performance of Vitek MS in the identification of yeasts showed an overall accuracy of 89.2%. Misidentifications or no identifications were more likely to occur in less-common species within each species complexes. In comparison, Autof MS 1000 system exhibited higher identification accuracy in all species complexes (>98% for all species with overall accuracy of 99.2%).

*C. albicans* is the most frequent cause of superficial and systemic candidiasis. Before the introduction of further genetic studies, its closely related species, *C. dubliniensis*, was commonly misidentified as *C. albicans* (Sullivan et al., 1995). However, unlike *C. albicans*, *C. dubliniensis* causes fewer cases of systemic candidiasis (Odds et al., 2007). In the present study, both Vitek MS and Autof MS 1000 correctly identified >96% of *C. albicans* isolates, and correctly assigned *C. dubliniensis* to species-level.

*C. parapsilosis* has become the second to fourth most prevalent *Candida* species worldwide, and even surpasses *C. albicans* in some geographic regions (Harrington et al., 2017; Tóth et al., 2019; Xiao et al., 2020). Before the introduction of molecular tests and further biochemical investigations, *C. orthopsilosis*, and *C. metapsilosis* used to be classified into the *C. parapsilosis* complex (Tavanti et al., 2005). In addition, *L. elongisporus*, which may be misidentified as *C. parapsilosis* by convientional methods, remains underestimated in its clinical significance (Lockhart et al., 2008; Al-Obaid et al., 2018). However, the incidence, pathogenicity, and drug resistance of species within the *C. parapsilosis* complex has been shown to be different (Neji et al., 2017; Xiao et al., 2020). Overall, there was a significant difference in the performance of Autof MS 1000 (99.0%) and Vitek MS (79.1%) in assigning *C. parapsilosis* complex isolates to the correct species-level. Of note, Vitek MS system exhibited significant limitations in correctly identifying *C. orthopsilosis* (accuracy 23.1%) and *C. metapsilosis* (accuracy 4.7%), while Autof MS 1000 correctly identified all 64 C*. metapsilosis* and 96.2% of *C. orthopsilosis* isolates.

*C. glabrata* has emerged as the second most common cause of invasive candidiasis in the United States (Pfaller and Diekema, 2007), while in China it ranks fourth among clinically invasive yeast infections (Xiao et al., 2015). Its phylogenetically related species, *C. nivariensis* and *C. bracarensis*, were previously identified as *C. glabrata* by routine identification methods (Alcoba-Flórez et al., 2005; Correia et al., 2006). However their clinical significance and epidemiological role in candidiasis is different (Bishop et al., 2008). In this study, both systems correctly identified 98.9% of *C. glabrata* isolates. Autof MS 1000 system correctly identified all three *C. nivariensis* and one *C. bracarensis* isolates. However, no *C. nivariensis* or *C. bracarensis* isolates were correctly identified by Vitek MS. Of note, *C. bracarensis* is not included in the Vitek MS database IVD 3.0.

The basidiomycetous yeasts of the *C. neoformans*-*C. gattii* species complex are the causative agents of cryptococcosis with different pathogenicity (Samarasinghe and Xu, 2018). *C. neoformans* is more common in AIDS patients, whereas infections caused by *C. gattii* are more often reported in immunocompetent patients (Kwon-Chung et al., 2015). Previously, conventional L-canavanine glycine bromothymol blue (CGB) agar was used to distinguish the two species (Chen et al., 2014). In this study, Vitek MS and Autof MS 1000 correctly assigned 97.4% and 99.7% of *C. neoformans* isolates to species level, respectively. However, in 10 C*. gattii* isolates, the Vitek MS came up with a “ no identification” result in three isolates and misidentified two isolates as *C. neoformans*. In contrast, Autof MS 1000 accurately identified 90% of the *C. gattii* isolates with only one isolate misidentified as *C. neoformans*.

*C. auris* has recently drawn much attention clinically due to its multidrug resistant characteristics and fast spread worldwide (Spivak and Hanson, 2018). *C. auris*, and its closely-related species *C. haemulonii*, *C. duobushaemulonii*, and *C. pseudohaemulonii*, are difficult to identify using standard laboratory methods (Muñoz et al., 2018). In the identification of this species complex, both Autof MS 1000 and Vitek MS performed well, with only one isolate of *C. duobushaemulonii* not identified by Vitek MS.

The principle of MALDI-TOF MS in microbial identification is based on using scoring algorithms to match analyzed spectra with reference spectra (Freiwald and Sauer, 2009; Jang and Kim, 2018). Regardless of the instruments used, distinct algorithms in the same platform result in different identification reports (Panagea et al., 2015; Leyer et al., 2017). For Autof MS 1000, just like MALDI BioTyper (Bruker, Billerica, MA), the database is based on an isolate-specific references approach, while for Vitek MS, it is based on a taxonomical group-specific approach (Cassagne et al., 2016). In this study, Autof MS 1000 performed better than Vitek MS in the identification of closely-related yeasts causing invasive fungal diseases. We suppose that differences in reference spectral databases and scoring algorithms of these two platforms may contribute to the performance discrepancies.

Our study has some limitations. We only evaluated the systems’ performance on closely-related yeast species commonly seen in patients, it is not clear whether similar results would have been obtained on other yeast and filamentous fungal species. On the other hand, Vitek MS v3.0 System has shown its excellent accuracy for the identification of filamentous fungi (Rychert et al., 2018). Therefore, further investigations are needed for validating the performance of Autof MS 1000 (compared to other widely-used MALDI-TOF MS systems), in the identifitication of a broader range of bacterial and fungal species.

## Conclusion

MALDI-TOF MS has previously proven to be valuable in the routine identification of yeast species. In this study, Autof MS 1000 exhibited higher identification accuracy than Vitek MS system in the identification of yeast isolates within different species complexes. The identification capacity of Vitek MS, especially in identifying less-common species within phylogenetically closely-related species complexes, still needs to be improved.

## Data Availability Statement

The raw data supporting the conclusions of this article will be made available by the authors, without undue reservation.

## Author Contributions

QY processed the experimental data, performed the analysis, drafted the manuscript, designed the tables, and revised the manuscript. MX, the main conceptual ideas and proof outline, performed the analysis and revised the manuscript. XF, GZ, YY, J-JZ, and S-MD contributed to sample preparation, performed MALDI-TOF MS identification. J-WC, YL, M-LZ, S-YY, J-JH, X-FC, and XH carried out the experiments, performed 16S rDNA sequencing identification. FK, TK, and Y-CX revised the manuscript, and Y-CX was involved in planning and supervised the work. All authors contributed to the article and approved the submitted version.

## Funding

This work was supported by a National Major Science and Technology Project (grant number 2018ZX10712001); Beijing Hospitals Authority Youth Programe (QML20190301); the Natural Science Foundation of China (81802042, 81971979, 81802049); Beijing Nova Program (Z201100006820127); Outstanding Young Talents Cultivation Program in Dongcheng District (2018 grant to MX); Beijing Key Clinical Specialty for Laboratory Medicine - Excellent Project (No. ZK201000).

## Conflict of Interest

The authors declare that the research was conducted in the absence of any commercial or financial relationships that could be construed as a potential conflict of interest.
